# Control of multidrug-resistant organisms

**DOI:** 10.7196/AJTCCM.2019.v25i1.009

**Published:** 2019-04-12

**Authors:** Richard Raine

**Affiliations:** Departments of Critical Care and Pulmonology, Groote Schuur Hospital, Cape Town, South Africa

## Editorial

Multidrug-resistant organisms (MDROs) are an increasing threat to hospitalised patients, and particularly to patients in an intensive care unit (ICU). Historically, organisms such as *Acinetobacter baumannii* and *Pseudomonas aeruginosa* have been difficult to eradicate, but owing to variable virulence these organisms are often ‘colonisers’ rather than obligate pathogens. Their presence is often a marker of long hospital stay and reduced immunity in a patient, with a variable contribution to increased mortality. The recent advent of carbapenem-resistant Enterobacteriaceae is far more concerning, as these organisms are often virulent and have resistance-transmission modes, such as plasmids, that allow easy transfer of resistance between organisms.

Many strategies to combat the spread of MDROs have been proposed and the World Health Organization has produced a guideline addressing these strategies.^[1]^ Unfortunately, many of these strategies are strongly recommended but are not accompanied by high-quality supportive evidence. The first recommendation involves implementing multimodal infection prevention and control (IPC) strategies, which include the use of IPC techniques that have proven effective in reducing hospital-acquired infections. The use of IPC bundles is included in this strategy.

As shown in the study by Aboshakwa *et al*.^[2]^ in this issue of AJTCCM, the use of bundles for ventilators, central-line insertion and maintenance and urinary catheter management is effective in reducing the prevalence of MDROs. Bundles require considerable staff training and commitment to compliance, and continuous monitoring of compliance is essential. Compliance rates of more than 80% have been shown to be effective.^[3,4]^

In addition to these interventions, the single most important – and most commonly neglected – component of IPC is hand hygiene. Hand hygiene that involves the use of alcohol-based handrub (and handwashing when appropriate) is central to preventing the spread of MDROs. Studies assessing hand hygiene compliance have shown baseline compliance rates of 34% on average, rising to 57% after interventions such as training, performance feedback and compliance monitoring.^[5]^ There are many reasons for poor compliance, but the harsh reality is that the simplest (and cheapest) intervention was not implemented effectively in nearly half of the studies reported. Other recommended interventions include contact precautions, patient isolation or cohorting, environmental cleaning and taking surveillance cultures from patients and environment.

However, alcohol-based hand hygiene remains simplest. Chlorhexidine is a common additive to locally available handrubs, but there are increasing concerns about chlorhexidine resistance, antiseptic stewardship and allergic reactions

The WHO has suggested an educational strategy that emphasises the five moments when hand hygiene should occur.^[6]^ These are:

1. Before touching a patient
2. Before a clean/aseptic procedure
3. After body fluid exposure risk
4. After touching a patient
5. After touching patient surroundings

The last moment in this list is the most commonly neglected. Any object in the close patient environment – including charts, equipment, furniture and mobile phones – is likely to be contaminated, and these are frequently handled without thought to hand hygiene.

There are a number of potentially expensive and intrusive recommendations to reduce transmission of MDROs. These range from the use of contact precautions (e.g. using a gown, apron and gloves for every patient contact) to single rooms for every patient in the ICU. Although the latter would require massive disruption of current services, it should be planned for any new facility. For now, however, hand hygiene remains the most effective intervention at existing facilities.
